# Acute and Chronic Changes in Myocardial Work Parameters in Patients with Severe Primary Mitral Regurgitation Undergoing Transcatheter Edge-to-Edge Repair

**DOI:** 10.3390/jcdd10030100

**Published:** 2023-02-25

**Authors:** Elena Galli, Pierre Hubert, Guillaume Leurent, Vincent Auffret, Vasileios Panis, Guillaume L’Official, Erwan Donal

**Affiliations:** Cardiology Department, University Hospital of Rennes, University of Rennes, LTSI-INSEMR, 35000 Rennes, France

**Keywords:** left ventricular remodeling, myocardial work, percutaneous mitral valve repair, primary mitral regurgitation

## Abstract

Background: The noninvasive assessment of myocardial work (MW) allows for the evaluation of left ventricular (LV) performance by considering the effect of LV afterload. This study aims to evaluate the acute and chronic impact of transcatheter edge-to-edge repair (TEER) on MW parameters and LV remodeling in patients with severe primary mitral regurgitation (PMR). Methods: A total of 71 patients (age: 77 ± 9 years, females: 44%) with moderate–to-severe or severe PMR (effective regurgitant orifice: 0.57 ± 0.31 cm^2^; regurgitant volume: 80 ± 34 mL; LV end-systolic diameter: 42 ± 12 mm) underwent TEER after a global assessment by the heart team. MW indices were evaluated before the procedure, at hospital discharge, and at 1-year follow-up. LV remodeling was described as the percentage variation in LVEDV between baseline and 1-year follow-up. Results: TEER caused an acute reduction in LVEF, global longitudinal strain (GLS), global MW index (GWI), work efficiency (GWE), and mechanical dispersion (MD) and a significant increase in wasted work (GWW). One year after the procedure, GLS, GWI, GWE, and MD recovered, whereas GWW remained significantly impaired. Baseline GWW (β = −0.29, *p* = 0.03) was an independent predictor of LV reverse remodeling at 1-year follow-up. Conclusions: In patients with severe PMR undergoing TEER, the acute reduction in LV preload causes significant impairment to all the parameters of LV performance. Baseline GWW was the only independent predictor of LV reverse remodeling, suggesting that a lower myocardial energetic efficiency in the context of chronic preload increase might impact the left ventricular response to mitral regurgitation correction.

## 1. Introduction

Severe primary mitral regurgitation (PMR) is associated with a chronic increase in left ventricular (LV) preload, which causes progressive LV dilatation and dysfunction [1]. The correct evaluation of LV function in patients with PMR is often difficult because the Frank-Starling mechanism allows the left ventricular ejection fraction (LVEF) to remain in the normal range despite the presence of a subtle alteration in LV performance [2]. Several studies have shown that an alteration in global longitudinal strain is a better indicator of LV dysfunction in patients with severe PMR [3,4] and is associated with outcome. Nevertheless, strain is also influenced by LV loading conditions, which might impact the evaluation of this parameter in patients with valvular heart disease. Russel et al. have recently demonstrated that the noninvasive assessment of myocardial work by pressure-strain loops (PSL) analysis allows for the evaluation of LV performance by considering the effects of LV load on the myocardium [5]. The aims of our work are, therefore, to (1) estimate different myocardial work parameters by PSL analysis in patients with severe PMR undergoing transcatheter edge-to-edge repair (TEER) by the clipping approach; (2) to assess the changes in myocardial work indices early after TEER and at 1-year follow-up (FU); (3) to identify the correlates of LV reverse remodeling at 1-year FU.

## 2. Materials and Methods

### 2.1. Population

Patients with severe PMR undergoing TEER (MitraClip^®^, Abbott structural, Santa Clara, CA, USA) according to recommendations [6] at the University Hospital of Rennes between September 2018 and September 2020 were retrospectively included in the study. Before TEER, all patients received optimized medical therapy in order to limit symptoms and volume overload. PMR was defined as the presence of excessive leaflets’ motion due to prolapse or flail, corresponding to the type II of Carpenter’s classification. All patients had a suitable acoustic window, allowing the assessment of echocardiographic parameters, and had no more than moderate concomitant valve disease. Clinical data, including age, gender, New York Heart Association (NYHA) functional class, and systolic and diastolic blood pressure, were assessed for each patient before TEER and at 1-year follow-up. Ischemic heart disease was defined by a history of previous myocardial infarction or significant coronary stenosis at coronary angiography. Patients with secondary atrial or ventricular mitral regurgitation were excluded from the study. The follow-up was available for all patients. The study was conducted following the “Good Clinical Practice” Guidelines in the Declaration of Helsinki and received Ethical Committee approval (CPP Sud Ouest et Outre Mer, CPP2021-03-036b/2021-A00972-39). All patients provided written informed consent before participation in the study. 

### 2.2. Echocardiography

All patients underwent transthoracic 2D-echocardiography using standard equipment (Vivid 9 or 95, GE Healthcare, Horten, Norway) equipped with a 3S or M5S 3.5-MHz transducer before TEER, at hospital discharge (3 ± 1 days after TEER), and 1-year FU FU (11 ± 2 months after TEER). Bidimensional, colour Doppler, pulsed-wave, and continuous-wave Doppler data were stored on a dedicated workstation for the offline analysis (EchoPAC, GEHealthcare, Horten, Norway). Cardiac dimensions and function, especially LV volumes, were measured according to current recommendations, and LVEF was calculated using the Simpson biplane method [7]. LV reverse remodeling was defined as the percentage variation in LV end-diastolic volume index (LVEDVi) between baseline and 1-year follow-up. PMR was quantified by the measurement of the effective regurgitant orifice (ERO) and the regurgitant volume (RV) using the proximal isovelocity surface area method. Total stroke volume (SV_tot_) was defined by the sum of the RV and stroke volume [8]. PMR was then classified as grade 1+ (mild: ERO < 0.20 cm^2^, RV < 30 mL), 2+ (mild-to-moderate: ERO: 20–20 mL, RV: 30–44 mL), 3+ (moderate-to-severe: ERO: 30–39 mL, RV 45–60 mL) or 4+ (severe: ERO ≥ 40 mL, RV ≥ 60 mL) as recommended [8]. As previously indicated in the EVEREST trial [9], procedural success was defined as the reduction of the PMR severity to 2 or less after TEER. To calculate LV global longitudinal strain (GLS), 2D greyscale images were acquired in standard apical four-, two- and three-chamber views at a frame rate of at least 60 frames/s. During offline analysis, a line was traced along the endocardium’s inner border in each of the three apical views on an end-systolic frame, and a region of interest was automatically defined between the endocardial and epicardial borders, with GLS automatically calculated from the strain in the three apical views [10]. For the purposes of this study, GLS was considered an absolute value. In patients with atrial fibrillation, we analyzed beats with approximately the same average heart rate as already done in previous larger trials [11]. Abnormal GLS was defined as <16.7% in men and <17.8% in women [12]. LV mechanical dispersion (MD) was assessed as the standard deviation of time to peak negative strain from 17-LV segments [13].

#### Assessment of Myocardial Work

Myocardial work (MW) and related indices were estimated using a vendor-specific module (EchoPAC Version 202, GE Vingmed Ultrasound, Horten, Norway) by the combination of LV strain data and the noninvasive estimation of the LV pressure. Briefly, peak systolic LV pressure is assumed to be equal to the peak arterial pressure recorded from the brachial cuff systolic pressure before the echocardiographic study. In all patients, systolic and diastolic blood pressure were measured in a supine position after five minutes of rest before the echocardiographic examination. The mean of three consecutive measures was retained for the purpose of the study. 

**Figure 1:**
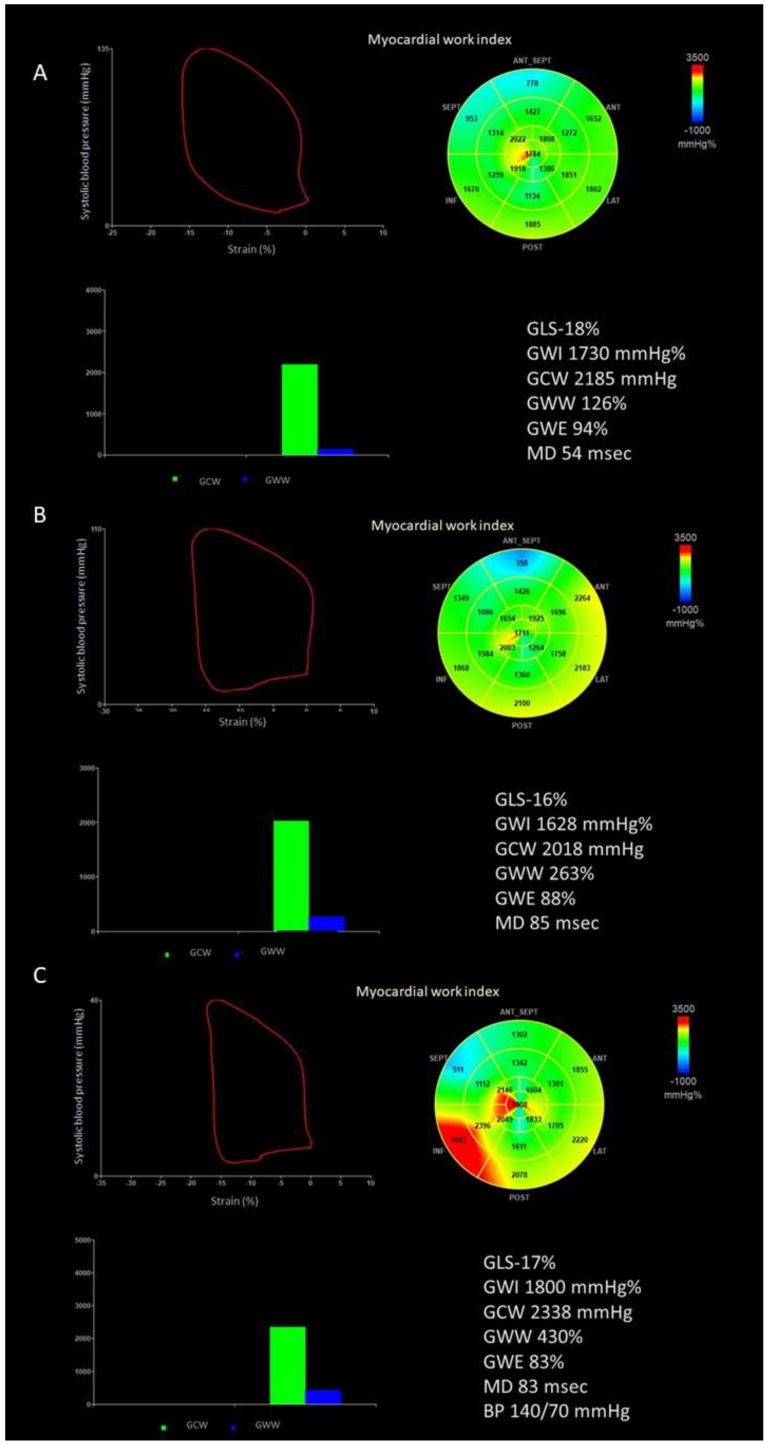
Example of the assessment of myocardial work indices. (**A**) Before transcatheter edge-to-edge repair of the mitral valve; (**B**) at hospital discharge; (**C**) at 1-year follow-up. BP, blood pressure; GLS, global longitudinal strain; GCW, global constructive work; GWE, global work efficiency; GWI, global work index; GWW, global wasted work; MD, mechanical dispersion.

The software then constructed a patient-specific LV pressure curve, adjusting the LV pressure curve to the duration of the isovolumic and ejection phases, defined by visual assessment of valvular timing events [5,14]. Strain and pressure data are synchronized using the R wave on ECG as a common time reference. Segmental LV work index is defined as the total work within the area of the LV pressure-strain loop, calculated from mitral valve closure to opening. Segmental constructive work is defined as the myocardial work during segmental shortening in systole and segmental lengthening during the isovolumic relaxation time. Segmental wasted work is defined as the work performed during lengthening in systole and shortening during isovolumic relaxation. Segmental work efficiency is defined as the ratio between myocardial constructive work and the sum of constructive work and wasted work. By averaging segmental work data for each LV segment, the global work index (GWI), constructive work (GCW), wasted work (GWW), and work efficiency (GWE) are estimated for the entire LV. The retained referral values are 1292–2505 mmHg% for GWI, 1582–2881 mmHg% for GCW, <198 mmHg% for GWW, and >90% for GWE [15]. An example of the assessment of myocardial work in patients before TEER, at hospital discharge, and at 1-year follow-up is depicted in Figure 1. Reproducibility of myocardial work assessment for the Corelab (CHU Rennes, iso 9001) has been previously reported [14]. The reproducibility of GWI, GCW and GWE for a subgroup of 20 patients included in this study is reported in Supplementary Figure S1.

### 2.3. Percutaneous Mitral Valve Repair

All procedures were performed under general anesthesia using transesophageal echocardiography and fluoroscopic guidance. Procedural success was defined as a noncomplicated placement of one or more Mitraclips^®^ (NT or NTW, Abbott structural, Santa Clara, CA, USA), coinciding with a per-procedural estimated MR reduction to a grade ≤ 2 [9].

### 2.4. Statistical Analysis

The normality of the distribution of continuous variables was tested by the Shapiro-Wilk test. Normally distributed variables were expressed as mean and standard deviation and compared using the paired sample Student’s *t*-test. Nonnormally distributed variables were expressed as the median and interquartile range and compared using Wilcoxon’s test. Categorical data are expressed as numbers and percentages and compared by the χ^2^-test. Correlations between variables were assessed by the linear regression analysis, and multiple linear regression was used to identify the best correlates of LV remodeling. The Bland-Altman plot and interclass correlation coefficient (ICC) were used to assess inter- and intra-echocardiographers’ variability in a subgroup of 20 patients. All statistical analyses were performed using Statistical Package for Social Science version 20.0 (SPSS Inc., Chicago, IL, USA) and MedCalc Software Ltd v20.111 (Ostend, Belgium). 

## 3. Results

### 3.1. Patients’ Characteristics

Seventy-one patients (mean age 77 ± 9 years, 44% females) with moderate–to-severe or severe PMR (ERO: 0.57 ± 0.31 cm^2^, regurgitant volume 80 ± 34 mL), and heart failure symptoms (NYHA 2.7 ± 7) were included in the study. Twenty-three (32%) patients had ischemic cardiomyopathy, and there was paroxysmal or persistent atrial fibrillation in 35 (49%) patients. All patients received optimized medical therapy before mitral TEER. TEER was successful in 68 (96%) patients. Only three patients had >2+ residual mitral regurgitation after TEER, corresponding to an ERO > 0.35 cm^2^ and RV > 0.45 mL. The severity of PMR decreased at hospital discharge and did not modify at 1-year follow-up. The main clinical characteristics of the patients are depicted in Table 1. 

**Figure 2:**
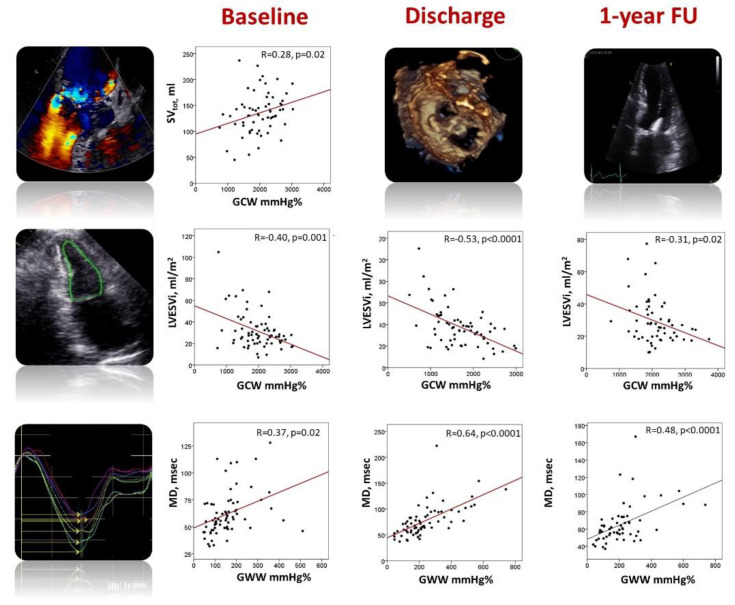
Relationship between myocardial work parameters, left ventricular total stroke volume, end-systolic volume, and mechanical dispersion before TEER (**left panels**), at hospital discharge (**central panels**) and 1-year follow-up (**right panels**). GCW, global constructive work; GWW, global wasted work; LVESVi, indexed left ventricular end-systolic volume; MD, mechanical dispersion; SVtot, total stroke volume.

Among the patients with preserved LVEF (n = 42, 59%), four (10%) had impaired GLS values. On the other hand, five (12%) and six (14%) patients had impaired GWI and GCW, and six (14%) patients had impaired GWW, whereas the alteration in GWE was evident in 10 (24%) patients. Before TEER, GCW and GWI showed an excellent correlation with GLS (r = 0.83 and r = 0.74, respectively; all *p* < 0.0001), a fair correlation with GWE (r = 62 and r = 0.50, respectively; *p* < 0.0001), a moderate correlation with systolic blood pressure (r = 0.44 and r = 0.56, respectively; both *p* < 0.0001), LVESVi (both r = −0.40; *p* < 0.0001), and a weak correlation with total stroke volume (r = 0.25, *p* = 0.04; 0.28, *p* = 0.02, respectively). GWW was well correlated with GWE (r = 0.73, *p* < 0.0001), modestly correlated with MD (r = 0.37, *p* = 0.002), GLS (r = −0.35, *p* = 0.004), and RF (r = −0.39, *p* = 0.002) (Figure 2, Table 2).

### 3.2. Acute and Chronic Impact of TEER on Left Ventricular Size and Function

**Figure 3:**
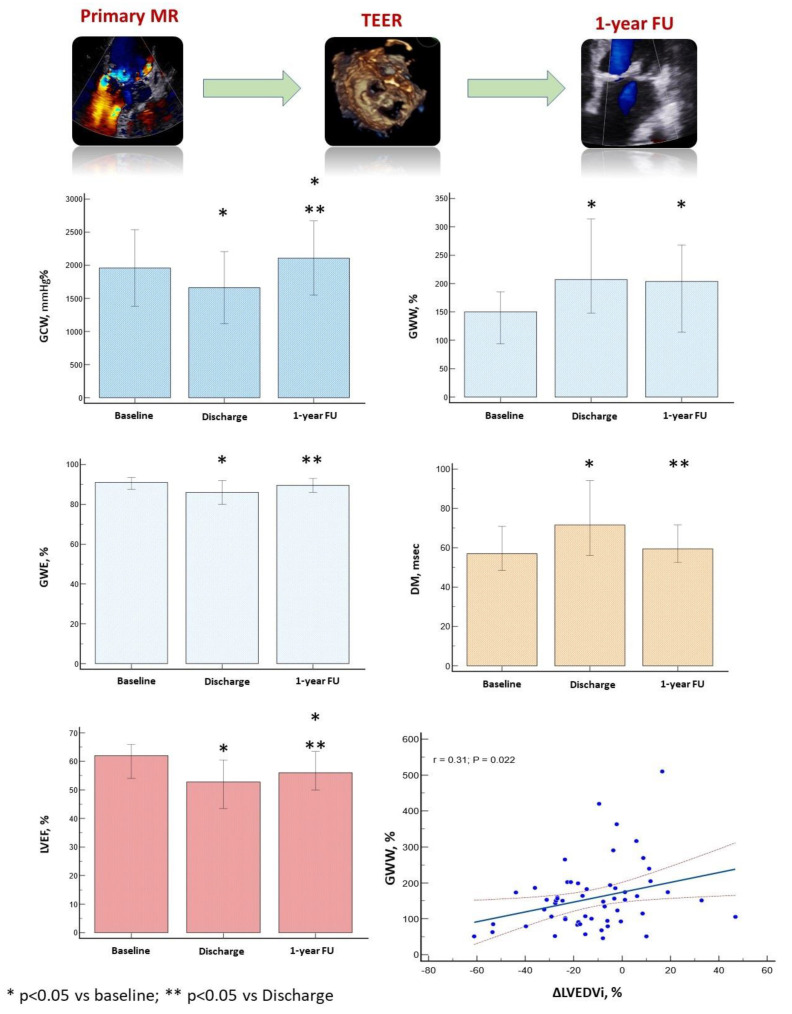
Variation of global constructive work, global work efficiency, global wasted work, left ventricular ejection fraction and mechanical dispersion from baseline to 1-year follow-up. Interestingly, baseline global wasted work was significantly associated with the left ventricular reverse remodelling at 1-year follow-up. Negative variation of the left ventricular end-diastolic volume corresponds to positive left ventricular reverse remodelling. Despite the significant variation of myocardial work parameters during the three-time points of the study, we observe a wide overlap of the standard deviation of these parameters and a significant dispersion of points at the linear correlation curve, which prevents the application of our results in clinical practice. GCW, global constructive work; GWE, global work efficiency; GWW, global wasted work; LVEF, left ventricular ejection fraction; MD, mechanical dispersion; ΔLVEDV_i_, percentage variation of the indexed left ventricular end-diastolic volume between baseline and follow-up.

The evolution of LV size and function before TEER, at hospital discharge, and 1-year follow-up is depicted in Table 3 and in Figure 3.

The acute reduction in LV preload was associated with a significant increase in LV end-systolic volume and a decrease in GLS, GWI, GCW, and GWE. On the other hand, GWW and MD significantly increased (Table 2, Graphical abstract). GWI and GCW remained significantly associated with GLS (r = 0.83 and r = 0.78, both *p* < 0.0001) and moderately correlated with GWE (r = 0.65 and r = 0.51; both *p* < 0.0001) and SBP (r = 0.50 and r = 0.63, both *p* < 0.0001). The strength of the correlation between GWI, GCW, and LVESVi increased (r = −0.51 and r = −0.53, both *p* < 0.0001). GWW was fairly correlated with GWE (r = 0.61, *p* < 0.0001) and GLS (r = −0.46, *p* < 0.0001). Interestingly, the strength of the correlation between GWW and MD increased (r = 0.64, *p* < 0.0001) (Figure 2, Table 2).

One year after TEER, LV size significantly decreased. GLS, GWI, GWE, and MD improved, reaching the baseline values. GCW increased above the baseline values, whereas GWW remained significantly reduced. Interestingly, LVEF improved compared to the post-clipping value but remained significantly altered compared to baseline values (Table 2, Figure 3). At 1-year FU, GWI and GCW were still well correlated with GLS (r = 0.72 and r = 0.70, respectively; both *p* < 0.0001) and GWE (r = 0.63 and r = 0.49, respectively; both *p* < 0.0001). They were moderately associated with LVESVi (r = −0.24, *p* = 0.04 and r = −0.31, *p* = 0.02, respectively) and SBP (r = 0.26, *p* = 0.04 and 0.25, *p* = 0.05). On the other hand, GWW was still well correlated with GWE (r = −0.86, *p* < 0.0001) and MD (r = 0.48, *p* < 0.0001) (Figure 2, Table 2). 

### 3.3. Predictors of Left Ventricular Remodeling 

One year after TEER, baseline LAVi and GWW were significantly correlated with the percentage reduction in LVEDVi. From the multivariable linear regression analysis, only GWW remained a significant predictor of LV reverse remodeling (β = 0.29, *p* = 0.03) (Table 4, Graphical abstract). 

Despite the significant variation in the myocardial work parameters during the three-time points of the study, we observe a wide overlap for the standard deviation of these parameters and a significant dispersion of points at the linear correlation curve, which prevents the application of our results in clinical practice. 

GCW, global constructive work; GWE, global work efficiency; GWW, global wasted work; LVEF, left ventricular ejection fraction; MD, mechanical dispersion; ΔLVEDV_i_, percentage variation in the indexed left ventricular end-diastolic volume between baseline and follow-up. 

## 4. Discussion

In this paper, we demonstrated that the noninvasive assessment of myocardial work: (1) is feasible in TEER candidates and (2) might contribute to explaining some of the mechanisms of the afterload mismatch and LV reverse remodeling observed after the correction of MR. 

### 4.1. Assessment of Left Ventricular Performance in Mitral Regurgitation

TEER is an effective procedure to treat severe PMR in patients who have a contraindication for conventional cardiac surgery and suitable valvular anatomy [6]. However, the assessment of myocardial function in the context of valvular heart disease remains highly challenging, and the effects of TEER on LV size and function are not established. Compared to LVEF and GLS, the noninvasive assessment of myocardial work has the merit of considering the effect of LV load on LV performance and allows for the indirect estimation of LV oxygen demand [5], which can be useful in patients with valvular heart disease. 

Previous studies have shown that a reduction in GLS might be observed in patients with severe primary mitral regurgitation and preserved LVEF and is associated with poor prognosis [2,3]. In our population, only 4% of patients with preserved LVEF had a con significant comitant reduction in GLS, whereas at least 12–24% of these patients showed a significant alteration in the myocardial work parameters, supporting the hypothesis that the noninvasive assessment of myocardial work allows for a more refined evaluation of LV performance. 

In these patients, the progressive LV dilatation due to the increased preload causes a significant increase in LV wall stress, which is directly proportional to LV systolic pressure and chamber size, according to the Laplace law [16]. The assessment of myocardial work that incorporates the measure of LV shortening/deformation and LV ejection impedance might provide a more careful assessment of LV performance. As a matter of fact, in our population, GCW—a measure of the work that is functional to LV shortening during systole and lengthening during LV isovolumic relaxation—was significantly correlated with total stroke volume but negatively correlated with the LVESVi.

### 4.2. Acute Changes in Left Ventricular Performance after TEER

The acute removal of the chronic overload condition through the clipping approach is associated with an immediate increase in stroke volume, an abrupt reduction in LV wall stress, and a decrease in systemic vascular resistance [17]. These aspects, together with the increase in LVESVi, can explain the significant decrease in all parameters of LV performance. Interestingly, MD and GWW significantly increased after TEER. The rise in MD after MR has already been described [18] and might result from decreased myocardial contractility due to afterload mismatch. Previous studies have shown that GWW is largely affected by the degree of LV mechanical dyssynchrony [5,19,20], which can explain the correlation existing between GWW and MD and its rise immediately after TEER. 

At 1-year follow-up, we observed a significant decrease in LV volumes and recovery in GCW, GWE, and MD. However, GWW remained significantly altered. The persistent increase in GWW might be attributable to the persistence of myocardial fibrosis [21,22], despite the correction of MR, and eventually counterbalance the increase in MWI and MCW, thus justifying the partial recovery in LVEF we observe in our population. 

### 4.3. Left Ventricular Reverse Remodeling after TEER

The determinants of LV remodeling after TEER are objects of debate [18,23,24,25]. The main studies conducted on the topic are heterogeneous, which makes the comparison with our survey quite difficult. Two previous publications on TEER have shown a correlation between GCW and LV systolic remodeling [25] or survival [26]. Nevertheless, these studies included patients with a high prevalence of secondary mitral regurgitation and severe LV dysfunction at baseline. In our population, GCW significantly increased 1 year after TEER, which can be attributed to a substantial reduction in LV load and an increase in vascular resistances. Nevertheless, only GWW was a significant predictor of LV remodeling at multivariable analysis. GWW is a measure of myocardial energy loss during the cardiac cycle and has been associated with LV remodeling in patients undergoing CRT [19,20]. GWW allows for the quantification of the work carried out by the LV, which does not contribute to LV ejection and might represent a measure of the LV contractile reserve [20]. Consequently, a lower LV energetic efficiency before TEER might indicate a poor LV response to the chronic hemodynamic burden of mitral regurgitation. This condition might contribute to increasing the sensitivity of the LV to afterload mismatch and negatively influence LV reverse remodeling. 

### 4.4. Clinical Implications Acute Changes in Left Ventricular Performance after TEER

The conventional assessment of LV performance is based on the evaluation of myocardial shortening and does not consider LV afterload. Mitral regurgitation is associated with a marked increase in LV preload. Nevertheless, in the presence of LV dilatation, both chamber enlargement and LV afterload contribute to the increased LV wall stress and oxygen demand [16]. The noninvasive assessment of MW might refine the evaluation of LV function in patients with severe PMR and contribute to the identification of factors associated with a deleterious response to mitral regurgitation correction. However, myocardial work parameters show a wide standard deviation/range. Moreover, the correlation between myocardial work and other variables, as depicted in Table 2, is often weak. This means that, at this stage, our results are not useful, at the individual level, to guide clinical management.

### 4.5. Limitations

This is a retrospective, monocentric, hypothesis-generating study, including a small group of patients with PMR. We decided to include patients with atrial fibrillation, which did not prevent the assessment of GLS and MW and is quite a common finding in patients with severe mitral regurgitation. In our study, the estimation of MW in patients with atrial fibrillation was made possible by choosing beats with approximately the same average heart rate, but it might be challenging in the case of extreme R-R variability.

The impact of atrial fibrillation ablation on LA and LV reverse remodeling and prognosis [27] has not been established in our study but might deserve specific analysis in this particular population of patients.

Eventual modification in medications during follow-up might potentially influence blood pressure and, therefore, the assessment of myocardial work. However, these modifications were not considered in this study, which might represent a bias for myocardial work analysis.

During follow-up, we focused on the assessment of LV volumes, and we did not assess LV diameter, so the impact of mitral TEER on LV diameters cannot be determined in our study.

The noninvasive assessment of MW is proposed by a vendor only (EchoPAC Version 202, GE Vingmed Ultrasound, Norway), and strain datasets coming from other vendors cannot be analyzed, which might represent a limitation for the wide application of MW in clinical practice. The estimation of the LV pressure curve from the systolic blood pressure assessed at an arm-cuff sphygmomanometer can be imperfect. Nevertheless, Hubert et al. have shown that small imprecisions in the noninvasive estimation of LV pressure do not affect the measure of myocardial work parameters [28]. The presence of LV fibrosis as a determinant of GWW was only inferred in our study. The systematic quantification of interstitial and diffuse fibrosis by cardiac MRI might provide further information on the severity of myocardial damage in PMR.

Patients included in the study had very severe cardiomyopathy and contraindication to conventional surgery. As a consequence, our results should be interpreted cautiously, particularly when transposed to a more classical population of patients undergoing surgery for severe PMR. Finally, the large variability of myocardial work parameters prevents their application in everyday practice to guide clinical management. However, our results might be useful on a population basis to disclose some of the mechanisms that are associated with LV remodeling and afterload mismatch in patients undergoing TEER.

## 5. Conclusions

The noninvasive assessment of myocardial work parameters is feasible in patients with PMR undergoing TEER and might contribute to underscoring the mechanisms of afterload mismatch observed acutely after a reduction in mitral regurgitation. Moreover, baseline GWW is an independent predictor of the entity of LV reverse remodeling at 1-year follow-up. Further studies are mandatory for confirming these preliminary results in a larger population of patients, including those undergoing surgical mitral valve repair/replacement.

## Figures and Tables

**Table 1:** Baseline clinical characteristics of the population.

	N = 71
Age, years	77 ± 9
Females, n (%)	31 (44%)
NYHA	2.7 ± 0.7
Arterial hypertension, n (%)	41 (58)
Diabetes, n (%)	5 (7)
Dyslipidemia, n (%)	33 (47)
Ischemic heart disease, n (%)	23 (32)
Chronic kidney failure, n (%)	18 (25)
Atrial fibrillation, n (%)	35 (49)
ACE-I/ARA-2, n (%)	33 (47)
Beta-blockers, n (%)	50 (70)
MRA, n (%)	11 (16)
Sacubitril/valsartan, n (%)	5 (7)
Diuretics, n (%)	62 (87)
Effective regurgitant orifice, cm^2^	0.57 ± 0.31
Regurgitant volume, mL	80 ± 34
LV end-diastolic diameter, mm	56 ± 9
LV end-systolic diameter, mm	42 ± 12
LVEF, %	60 ± 11
LVEF ≥ 60%	42 (59)

ACE-I, angiotensin, converting enzyme inhibitors; ARA, angiotensin receptor antagonist; MRA, mineralocorticoid receptor antagonists; LV, left ventricle; LVEF, left ventricular ejection fraction; NYHA, New York Heart Association functional class.

**Table 2:** Main correlates of myocardial work indices at baseline, hospital discharge, and 1-year follow-up.

**Baseline**	**GWI**		**GCW**		**GWW**		**GWE**	
	**r**	***p*-Value**	**r**	***p*-Value**	**r**	***p*-Value**	**r**	***p*-Value**
LVEDVi	−0.11	0.35	−0.16	0.18	−0.03	0.79	−0.15	0.22
LVESVi	−0.40	0.001	−0.40	0.001	0.12	0.32	−0.45	<0.001
GLS	0.83	<0.001	0.74	<0.001	−0.35	0.004	0.67	<0.001
ERO	−0.14	0.25	−0.21	0.09	−0.19	0.14	0.03	0.81
RV	0.06	0.63	0.04	0.76	−0.15	0.25	0.07	0.57
RF	0.03	0.80	−0.15	0.23	−0.39	0.002	0.13	0.29
SV_tot_	0.25	0.04	0.28	0.02	0.05	0.72	0.07	0.61
SBP	0.44	<0.001	0.56	<0.001	0.34	0.005	−0.03	0.78
DBP	0.08	0.47	0.009	0.94	0.29	0.02	−0.26	0.03
GCW	0.92	<0.001	-	-	−0.02	0.89	0.50	<0.001
GWW	−0.23	0.05	−0.02	0.89	-	-	−0.73	<0.001
GWE	0.62	<0.001	0.50	<0.001	−0.73	<0.001	-	-
MD	−0.17	0.16	−0.10	0.41	0.37	0.02	−0.52	<0.001
**Discharge**	**GWI**		**GCW**		**GWW**		**GWE**	
	**r**	***p*-Value**	**r**	***p*-Value**		***p*-Value**		***p*-Value**
LVEDVi	−0.22	0.08	−0.26	0.04	−0.08	0.52	−0.03	0.79
LVESVi	−0.51	<0.001	−0.53	<0.001	0.1	0.42	−0.33	0.006
GLS	0.83	<0.001	0.78	<0.001	−0.46	<0.0001	0.64	<0.001
SBP	0.50	<0.001	0.63	<0.001	0.22	0.06	0.15	0.22
DBP	0.17	0.17	0.22	0.08	0.08	0.54	−0.01	0.91
GCW	0.94	<0.001	-	-	−0.12	0.34	0.51	<0.001
GWW	−0.36	0.002	0.12	0.34	-	-	−0.61	<0.001
GWE	0.65	<0.001	0.51	<0.001	−0.61	<0.001	-	-
MD	−0.32	0.005	−0.20	0.11	0.64	<0.001	−0.39	0.001
**1-Year FU**	**GWI**		**GCW**		**GWW**		**GWE**	
	**r**	***p*-Value**	**r**	***p*-Value**		***p*-Value**		***p*-Value**
LVEDVi	−0.07	0.59	−0.15	0.27	−0.11	0.41	0.006	0.97
LVESVi	−0.24	0.05	−0.31	0.02	−0.09	0.51	0.07	0.61
GLS	0.72	<0.001	0.70	<0.001	−0.29	0.03	0.52	<0.001
SBP	0.26	0.04	0.25	0.05	0.14	0.32	0.02	0.91
DBP	0.26	0.05	0.27	0.04	0.12	0.37	0.01	0.92
GCW	0.95	<0.001	-	-	−0.09	0.48	0.49	<0.001
GWW	−0.28	0.03	−0.09	0.48	-	-	−0.86	<0.001
GWE	0.63	<0.001	0.49	<0.001	−0.86	<0.001	-	-
MD	−0.26	0.05	−0.19	0.16	0.48	<0.001	−0.53	<0.001

ERO, effective regurgitant orifice; FU, follow-up; GCW, global constructive work; GWE, global work efficiency; GWI, global work index; GWW, global wasted work; GLS, global longitudinal strain; LAVi, indexed left atrial volume; LVEDVi, indexed left ventricular end-diastolic volume; LVEF, left ventricular ejection fraction; LVESVi; RV, regurgitant volume; MD, mechanical dispersion; RF, regurgitant fraction; RV, regurgitant volume.

**Table 3:** Echocardiographic parameters at baseline, at hospital discharge after TEER, and 1-year follow-up.

	Baseline	Hospital Discharge	1-Year FU
LVEF, %	62 (54–66)	43 (35–61) *	56 (50–63) *^,^**
GLS, %	16 ± 5	12 ± 5 *	15 ± 4 **
LVEDVi, mL/m^2^	73 ± 24	70 ± 22	64 ± 22 *^,^**
LVESVi, mL/m^2^	27 (21–35)	36 (25–44) *	26 (19–36) **
SV, mL	53 (42–64)	60 (46–83) *	59 (40–69) *
LAVi, mL/m^2^	73 (55–87)	74 (54–85)	67 (55–89)
E/e’	13 ± 4	16 ± 7	15 ± 11
PAPs, mmHg	54 ± 12	44 ± 12 *	44 ± 19 *
TAPSE, mm	20 ± 5	20 ± 5	20 ± 4
SBP, mmHg	119 (103–136)	118 (104–132)	124 (111–137) *^,^**
DBP, mmHg	77 ± 11	66 ± 16 *	75 ± 11 **
GWI, mmHg%	1677 ± 551	1308 ± 524 *	1707 ± 527 **
GCW, mmHg%	1985 ± 562	1696 ± 567 *	2112 ± 555 *^,^**
GWW, mmHg%	150 (93–185)	208 (148–312) *	202 (116–267) *
GWE, mmHg%	91 (88–94)	87 (80–92) *	90 (86–93) **
MD, msec	57 (49–71)	72 (58–93) *	59 (52–72) **

* *p* < 0.05 vs. Baseline; ** *p* < 0.05 vs. discharge. DBP, diastolic blood pressure; GCW, global constructive work; GWE, global work efficiency; GWI, global work index; GWW, global wasted work; GLS, global longitudinal strain; LAVi, indexed left atrial volume; LVEDVi, indexed left ventricular end-diastolic volume; LVEF, left ventricular ejection fraction; LVESVi, indexed left ventricular end-systolic volume; MD, mechanical dispersion; PAPs, systolic pulmonary artery pressure; SBP, systolic blood pressure; SV, stroke volume; TAPSE, tricuspid annular plane systolic excursion.

**Table 4:** Predictors of left ventricular reverse remodeling at 1-year follow-up.

	Univariable Analysis		Multivariable Analysis	
	β	*p*-Value	β	*p*-Value
LVEDVi, mL/m^2^	−0.23	0.09		
LVESVi, mL/m^2^	−0.06	0.65		
LVEDV, mm	0.18	0.19		
LVESV, mm	0.05	0.72		
LVEF, %	−0.20	0.14		
GLS, %	−0.15	0.25		
LAVi, mL/m^2^	−0.26	0.05	−0.24	0.07
E/e’	0.10	0.58		
ERO, cm^2^	−0.19	0.17		
RV, mL	−0.23	0.09		
GWI, mmHg%	−0.09	0.48		
GCW, mmHg%	−0.04	0.76		
GWW, mmHg%	0.31	0.02	0.29	0.03
GWE, mmHg%	−0.19	0.17		

ERO, effective regurgitant orifice; GCW, global constructive work; GWE, global work efficiency; GWI, global work index; GWW, global wasted work; GLS, global longitudinal strain; LAVi, indexed left atrial volume; LVEDVi, indexed left ventricular end-diastolic volume; LVEF, left ventricular ejection fraction; LVESVi; RV, regurgitant volume.

## Data Availability

Data sharing is not applicable to this article due to privacy.

## Supplementary Materials

The following supporting information can be downloaded at: https://www.mdpi.com/article/10.3390/jcdd10030100/s1, Figure S1: Bland–Altman plot analysis and interclass correlation coefficients for inter-observer and intra-observer agreement for left ventricular end-diastolic volume (**A** and **B**), left ventricular end-systolic volume (**C** and **D**), global work index (**E** and **F**), global constructive work (**G** and **H**), and global wasted work (**I** and **J**).
