# Supplementary material for: Acute and Chronic Changes in Myocardial Work Parameters in Patients with Severe Primary Mitral Regurgitation Undergoing Transcatheter Edge-to-Edge Repair
Source: J Cardiovasc Dev Dis. 2023 Feb 25;10(3):100. doi: 10.3390/jcdd10030100 (PMC10051684; doi:10.3390/jcdd10030100)
Supplement: Supplementary file 1 [file jcdd-10-00100-s001.zip › jcdd-2227948-supplementary.pdf]

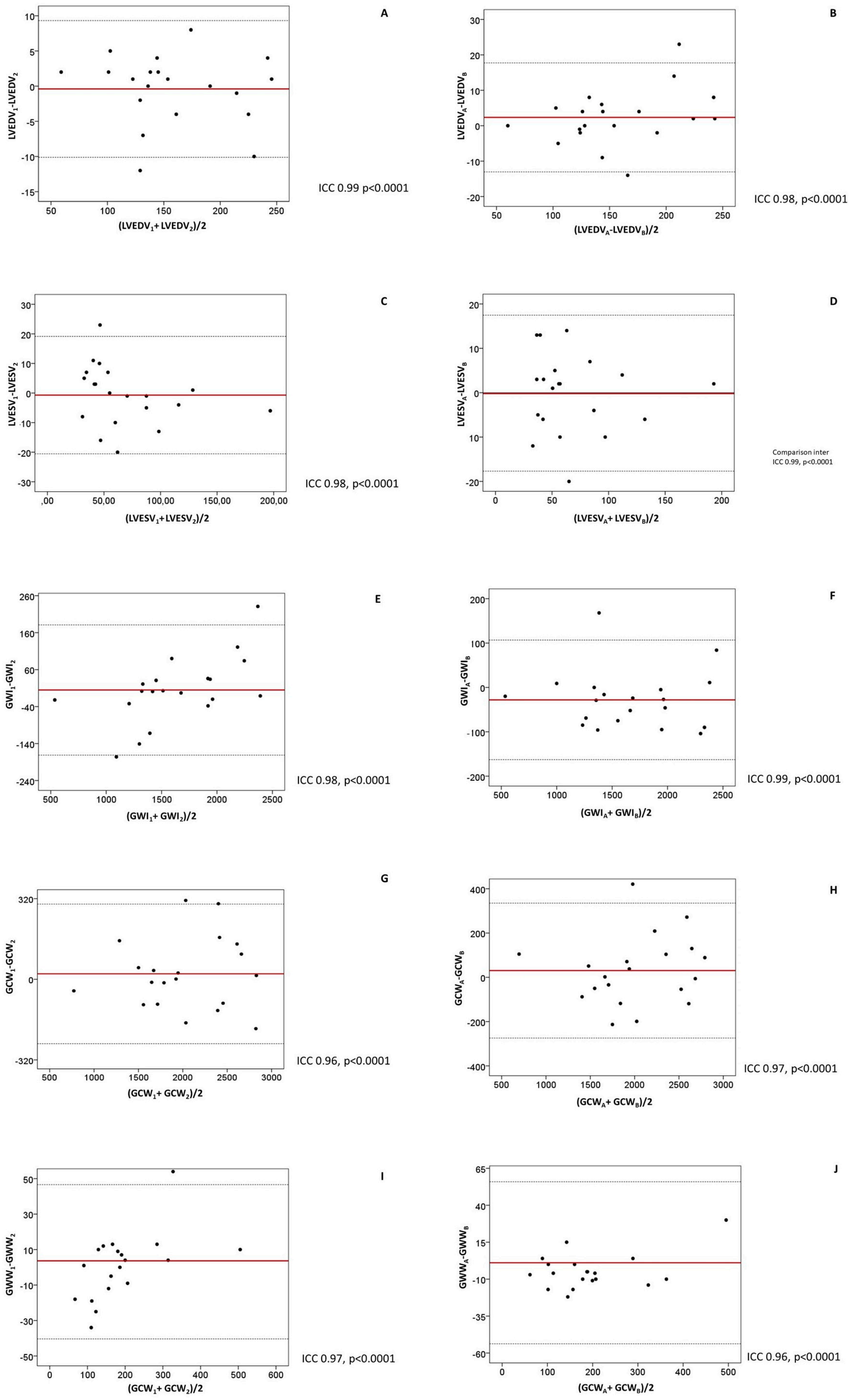

Figure S1. Bland–Altman plot analysis and interclass correlation coefficients for inter-observer and intra-observer agreement for left ventricular end-diastolic volume (**A** and **B**), left ventricular end-systolic volume (**C** and **D**), global work index (**E** and **F**), global constructive work (**G** and **H**), and global wasted work (**I** and **J**). A and B indicate measures performed by two independent echocardiographers to test inter-observed agreement. Numbers 1 and 2 indicate two measures performed by the same echocardiographer to test intra-observed agreement. ICC, interclass correlation coefficient; GCW, global constructive work; GWW, GWI, global work index; global wasted work; LVEDV, left ventricular end-diastolic volume; LVESV, left ventricular end-systolic volume.
